# Identification of a Novel Geminivirus in *Fraxinus rhynchophylla* in Korea

**DOI:** 10.3390/v13122385

**Published:** 2021-11-28

**Authors:** Aamir Lal, Yong-Ho Kim, Thuy Thi Bich Vo, I Gusti Ngurah Prabu Wira Sanjaya, Phuong Thi Ho, Hee-Seong Byun, Hong-Soo Choi, Eui-Joon Kil, Sukchan Lee

**Affiliations:** 1 Department of Integrative Biotechnology, Sungkyunkwan University, Suwon 16419, Korea; aamirchaudhary43@gmail.com (A.L.); bichthuy251188@gmail.com (T.T.B.V.); gusti.prabu20@gmail.com (I.G.N.P.W.S.); hophuongk59sinhhoc@gmail.com (P.T.H.); 2 Crop Protection Division, National Institute of Agricultural Sciences, Rural Development Administration, Jeonju 55365, Korea; ksnrn93@gmail.com (Y.-H.K.); hsbyun73@korea.kr (H.-S.B.); 3 Department of Plant Medicals, Andong National University, Andong 36729, Korea

**Keywords:** geminivirus, begomovirus, Fraxinus symptomless virus, Fraxinus rhynchophylla

## Abstract

*Fraxinus rhynchophylla*, common name ash, belongs to the family *Oleaceae* and is found in China, Korea, North America, the Indian subcontinent, and eastern Russia. It has been used as a traditional herbal medicine in Korea and various parts of the world due to its chemical constituents. During a field survey in March 2019, mild vein thickening (almost negligible) was observed in a few ash trees. High-throughput sequencing of libraries of total DNA from ash trees, rolling-circle amplification (RCA), and polymerase chain reaction (PCR) allowed the identification of a *Fraxinus symptomless virus.* This virus has five confirmed open reading frames along with a possible sixth open reading frame that encodes the movement protein and is almost 2.7 kb in size, with a nonanucleotide and stem loop structure identical to begomoviruses. In terms of its size and structure, this virus strongly resembles begomoviruses, but does not show any significant sequence identity with them. To confirm movement of the virus within the trees, different parts of infected trees were examined, and viral movement was successfully observed. No satellite molecules or DNA B were identified. Two-step PCR confirmed the virion and complementary strands during replication in both freshly collected infected samples of ash tree and *Nicotiana benthamiana* samples agro-inoculated with infectious clones. This taxon is so distantly grouped from other known geminiviruses that it likely represents a new geminivirus genus.

## 1. Introduction

*Fraxinus*, commonly known as ash tree, is an important member of the *Oleaceae* family found in North America, northeast Asia, east and western France, China, Korea, eastern Russia, Pakistan, India, and Afghanistan [1,2,3,4]. Chemically, *Fraxinus* plants contain various secoiridoids, phenylethanoids, flavonoids, coumarins, and lignans, so they are considered to have diverse biological and pharmacological activities [5,6]. Their immense range of pharmacotherapeutic properties, such as anticancer, anti-inflammatory, antioxidant, antimicrobial, and neuroprotective, make them highly valuable. In addition, their bioactive phytochemicals and secondary metabolites can be exploited as effective antiaging agents in the cosmetics business [1,2,5,7].

Among *Fraxinus* species, *Fraxinus rhynchophylla*, our target study, also known as East Asian ash, grows mainly in moist, fertile soils on hillsides and in river valleys in Korea, China, and Japan (https://species.nibr.go.kr, accessed on 29 September 2021). *F. rhynchophylla* regenerates and dominates naturally after thinning in Korea, promoting the restoration of native hardwood forests [8]. It serves as a wildlife habitat, helps to stabilize stream banks, and contributes organic matter to the forest. In addition to the medicinal benefits of its bark, its wood is used to manufacture furniture, sports equipment, and tool handles owing to its hard, dense, but elastic properties [2,6,9].

Few viruses have been reported as infectious agents in *Fraxinus* species. To date, only RNA viruses, namely, *Arabis mosaic virus*, *cherry leaf roll virus*, *tomato ringspot virus* and *tobacco ringspot virus* (genus: *Nepovirus*; family: *Sicoviridae*); *tobacco necrosis virus* (genus: *Alphanecrovirus*; family: *Tombusviridae*); *tobacco mosaic virus* (genus: *Tobamovirus*; family: *Virgaviridae*); and *white ash mosaic virus* (unclassified) [10,11,12,13,14,15,16,17,18,19], have been documented. To our knowledge, no plant-infecting DNA viruses have ever been reported to infect *Fraxinus* species.

High-throughput sequencing (HTS) technologies have revolutionized systems for detecting viruses [20,21]. This has resulted in a significant increase in the identification of novel viruses across ecosystems, as well as a broadening of our understanding of the diversity of plant-infecting viruses. Among plant virus families, the largest number of new and divergent viruses has been discovered through HTS in *Geminiviridae* [22]. Approximately 12 new genera discovered mainly through HTS have been classified recently, extending the nine recognized geminivirus genera to fourteen: *Becurtovirus*, *Begomovirus*, *Capulavirus*, *Citlodavirus*, *Curtovirus*, *Eragrovirus*, *Grablovirus*, *Maldovirus*, *Mastrevirus*, *Mulcrilevirus*, *Opunvirus*, *Topilevirus*, *Topocuvirus*, and *Turncurtovirus* [23,24,25,26]. Many of these genera have been classified based on viruses discovered through large-scale HTS-based virus discovery projects [27,28,29,30,31,32,33,34].

Geminiviruses are circular single-stranded DNA genomes encapsidated in twinned icosahedral particles and encode up to seven genes that are bi-directionally transcribed [24,35]. Among these seven genes, replication-associated protein gene (rep) and a capsid protein gene (cp) are detectably conserved across all of these divergent lineages [36,37]. Few genes are found conserved across the few genera within the family *Geminiviridae*, i.e., replication enhancer protein gene (ren), a C4 gene (which encodes a symptom determinant and/or a silencing suppressor), and a transactivation protein gene (trap), are possibly conserved in genera: *Begomovirus*, *Curtovirus*, *Eragrovirus*, *Topocuvirus*, and *Turncurtovirus* [24,36,37,38]. Movement protein genes (mp) are present in all known geminivirus genomes except in few recently reported geminiviruses [39].

Geminiviruses mainly cause severe economic losses in a variety of crops (i.e., tomato, maize, cotton, cassava, and bean plants) [36,40,41,42], but newly discovered geminiviruses appeared to produce either no symptoms or only mild ones in their host species [23,29,43]. Geminiviruses are transmitted by a range of insect vectors in the order Hemiptera [36,40]. In the past, geminiviruses as pathogens of cultivated plants were primarily focused on but recent reports of new virus species including geminiviruses as causative agents in various new hosts from natural ecosystems have caught more attention of plant virologists towards the emergence of new crop pathogens, especially geminiviruses from natural ecosystems [22,23,44,45,46,47,48,49,50].

Here, we describe the characterization of a novel geminivirus found to infect *Fraxinus* species, namely, *F. rhynchophylla*, in Korea. The virus was shown to exhibit a separate grouping during phylogenetic analysis and was thus named: *Fraxinus symptomless virus* (FSMV). Infectivity assays involving *Nicotiana benthamiana* confirmed the asymptomatically infection of FSMV.

## 2. Materials and Methods

### 2.1. Sample Collection and Processing

**Figure 1:**
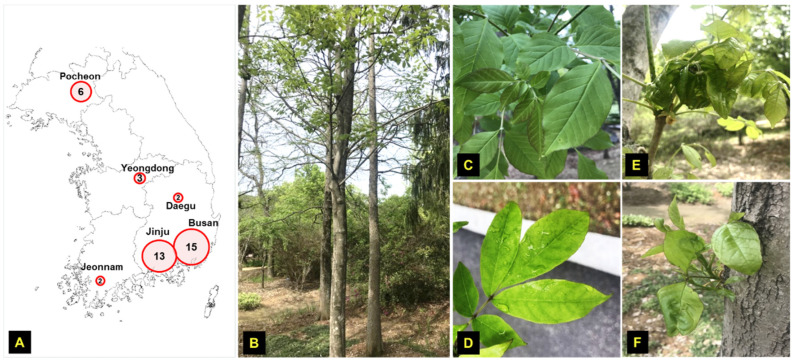
Ash tree (*F. rhynchophylla*) samples collection from various locations in Korea. (**A**) Targeted sites and the number of samples collected from each site. (**B**) Ash tree investigated in our study and the leaf samples collected from (**C**) Jinju, (**D**) Busan, (**E**) Pocheon, and (**F**) Yeongdong, respectively.

A total of 41 *F. rhynchophylla* plant samples from various regions of Korea were collected in different time periods (Table 1). All samples were asymptomatic and collected: Jinju (*n* = 4 in March 2019; *n* = 8 in September 2019), Busan (*n* = 9 in October 2019; *n* = 6 in May 2020), Pocheon (*n* = 6 in September 2019), Jeonnam (*n* = 2 in September 2019), Yeongdong (*n* = 3 in September 2019), and Daegu (*n* = 2 in September 2019) (Figure 1). No insects were found or collected from any of these 41 plants. All samples were stored at −20 °C until processing. All leaf samples were sterilized by using 70% ethanol for 20–30 s and allowed to dry off from the ethanol with the air flow under the fume hood. Total DNA was extracted from leaf tissue samples using either a Viral Gene-Spin Viral DNA/RNA Extraction Kit (iNtRON Biotechnology) or a cetyl trimethylammonium bromide (CTAB)-based extraction protocol, following the manufacturer’s instructions [51]. Total DNA from each sample was used in RCA reaction with the TempliPhi™ kit (GE Healthcare, Chicago, IL, USA), as described by Shepherd et al. [52].

### 2.2. HTS and Genome Assembly

Aliquots of the RCA product of two samples collected from the Jinju sample (J1 & J2) in March 2019 were sequenced on an Illumina HiSeq 4000 platform (paired end 2 × 100 bp) at Macrogen Inc. (Seoul, Korea). Raw reads were de novo assembled using SPAdes v.3.12.0 [53] and the resulting contigs were analyzed using BLASTx [54] against a GenBank viral RefSeq protein database [55]. PCR using abutting primers (TF2 5′-AGT GTT GGA CTC GAA TCC AGA A-3′ and TR2 5′- CTG GAC AGA CGA CGA ATC CA-3′) was processed following the manufacturer’s thermal cycling condition recommendations to recover potentially full-length virus genomes from plant samples (J1, J2, J5, J6 from Jinju and P2, P3, P4 from Pocheon). Amplicons were resolved in 1% agarose gel and those with target size of approximately 2.7 kb (the expected size range of geminivirus genomes) were excised, gel-purified, and cloned in the pGEM-3Zf (+) vector (Promega, Madison, WI) and sequenced by a commercial sequencing service (Macrogen, Seoul, Korea) followed by the sequence analysis in the NCBI Basic Local Alignment Search Tool (BLASTn) [54].

### 2.3. Detection of the Novel Virus in *F. rhynchophylla*

We selected a total of 41 DNA extracts representing different geographic areas and provinces across South Korea (Table 1). First, only four samples from Jinju (JI–J4) were processed. The DNA extracts were recovered from plant tissues consisting of leaf petioles and small portions of twigs, using the CTAB method. Based on full length sequence, new specific primers (Ash_Gemini_2F 5′-CCA CGT GTC ATC ATC TTA GG-3′ and Ash_Gemini_2R 5′- TAGTCCCGGTCAATTTCTTG-3′) of product size 737 bp, were designed for easy detection purpose and were mainly used for detection in all samples (Supplementary Table S1). PCR was processed following standard amplification conditions: denaturation at 94 °C (3 min), and then 35 cycles of 30 s at 94 °C, 30 s at 58 °C, and 1 min at 72 °C, followed by final extension at 72 °C (5 min). Amplicons were excised and sequenced as mentioned in Section 2.2. RCA followed by the digestion through restriction enzymes: *Kpn*I, *Pst*I, and *Bam*HI, and PCR with universal betasatellite [56], alphasatellite primers [57], and DNA-B primers [58] (Supplementary Table S1) attempted to detect the associated components, i.e., satellite molecules or DNA B (Supplementary Figure S1).

Leaf tissue samples were collected from three different sites of ash trees (B1 *–B6 *) and processed by PCR using Ash_Gemini_2F/R primers to confirm virus movement and infectivity in all parts of the trees.

### 2.4. Genome Organization and Homology Searches for Genes

PCR products obtained through conventional Sanger sequencing were assembled using multiple sequence alignment by Florence Corpet (MultAlin) [59]. Open reading frames (ORFs) were identified with ORFfinder (https://www.ncbi.nlm.nih.gov/orffinder/, accessed on 29 September 2020), and conserved domains were characterized using BLASTx and PLACE [54,60]. Identity matrices were obtained using the MUSCLE option in SDT v1.2 [61]. Alignments for nucleotide and amino acid homology were performed with the MUSCLE algorithm embedded in MEGA7 [62]. Phylogenetic relationships among members of geminivirids were evaluated using full-length nucleotide sequences with the neighbor-joining method in MEGA7 program with 1000 bootstrap replicates, as already described by the ICTV Taxonomy study group [63]. Maximum-likelihood phylogenetic trees were inferred from all genes of representative nucleotide isolate sequences of viruses from genera in the family *Geminiviridae.*

### 2.5. Attempts at Further Characterization by Southern Blot Hybridization

Southern hybridization analysis was conducted to confirm the viral replication of FSMV in the samples using the modified method from Southern et al. [64,65]. Total DNA (15 μg) isolated from 2 ash tree tissues samples from each location (Table 1) was loaded on 1% agarose gel followed by the depurination, denaturation, and neutralization steps and transferring the DNA loaded on the gel to a positively charged nylon membrane (Hybond-N+ membrane, GE Healthcare Life Sciences, Waukesha, WI, USA) using the capillary transfer method for up to 16 h and the transferred DNA was linked covalently to the nylon membrane using an ultraviolet crosslinker (UVC 500 crosslinker, GE Healthcare Life Sciences, Waukesha, WI, USA). The FSMV DNA-A (2.7 kb) was amplified from J1, B6, P4, and JM2 (Table 1) with the TF2/R2 primer set, was gel purified, and labeled with [α-32P] dCTP using the Rediprime II Random Primer Labeling System (GE Healthcare Life Sciences). Hybridization was conducted at 65 °C for 16 h. After washing, the membrane was then exposed to X-ray film (Kodak, Rochester, NY, USA) for approximately 48 h in a −70 °C freezer.

### 2.6. Strand-Specific PCR for Virus Detection

Strand-specific amplification method introduced by Rodríguez-Negrete using virion-sense- and complementary-sense-specific primer sets was conducted with slight modifications to detect the virus in the samples, i.e., J1, B6, P4, and JM2 [66,67]. In the first step, extension reactions of single-stranded viral templates with T4 DNA polymerase (TaKaRa, Japan) and viral-specific primers OCS-TAG or OVS-TAG were performed for strand-specific amplification followed by the purification through QIA quick PCR Purification Kit (Qiagen) (Supplementary Table S1). In the second step, 2 μL product of the first-strand reaction was mixed with 10 μL of 2X AccuPower PCR Master Mix (Bioneer), 1 μL of 10 pM specific primers (TAG, OVS, or OCS), and 6 μL of nuclease-free water following the manufacturer’s protocol and reacted for one cycle at 95 °C for 30 s, and then 40 cycles at 95 °C for 10 s, 60 °C for 15 s, and 72 °C for 20 s in a T100 thermal cycler (Bio-Rad, Hercules, CA, USA).

### 2.7. Construction of Infectious Clone of FSMV

Infectious clone (1.1 mer) of FSMV was constructed to check its infectivity in the host plants. Two partial genomes containing restriction sites at the edge were amplified using primer sets designed based on the sequence of FSMV and ligated into the pGEM-T Easy vector (Promega, Madison, WI, USA) using the TA cloning technique, in accordance with the manufacturer’s instructions followed by the sequencing (Macrogen, Korea) and restriction digestion with specific enzymes. These two partial genomes were introduced into the pCAMBIA1303 vector and first transformed into competent *Escherichia coli* strain DH5α using the heat shock method and then transformed into GV3101 *Agrobacterium* strains and confirmed by both enzyme digestion and colony PCR with the detection primer sets.

### 2.8. Agro-Inoculation with the FSMV Infectious Clone

*Nicotiana benthamiana* plants were planted in a growth chamber at Sungkyunkwan University, Suwon, Korea. Approximately 4-week-old *N. benthamiana* plants of similar sizes were selected to check the infectivity. *Agrobacterium* GV3101 strains (both transformed and untransformed) were cultured in LB broth in the presence of pCAMBIA1303 selection antibiotic, namely, kanamycin (50 mg/L), and strain-specific selection antibiotics, namely, gentamycin and rifampicin (50 mg/L), at 28℃ with agitation for 30 h (until the OD value at 600 nm was 0.8–1.0). Agro-inoculation was performed by the pin-pricking method [68]. Leaf tissue samples were collected from mock and infected plants 28 days post-inoculation (dpi) to check the infectivity through PCR processing using Ash_Gemini_2F/2R primers. Vector-specific primers were also used to make sure of the detection of the virus itself instead of pCAMBIA1303 plasmid containing the virus in different parts of the plant.

## 3. Results

### 3.1. HTS Results

HTS of the two DNA libraries yielded 74,391,351 raw paired reads. A total of 54,465 contigs were obtained from the libraries. BLASTx search of these contigs indicated the presence of virus-derived DNAs with an identity with *Geminiviridae* members. Pairwise alignment of putative DNA-A-like contigs revealed that DNA-A does not exhibit a greater identity with the reference viruses cited in the literature. A more accurate analysis of the sequence of the contig disclosed five ORFs in a circular pattern very similar to the findings in the genus *Begomovirus* of the family *Geminiviridae.*

### 3.2. Virus Detection through PCR

**Figure 2:**
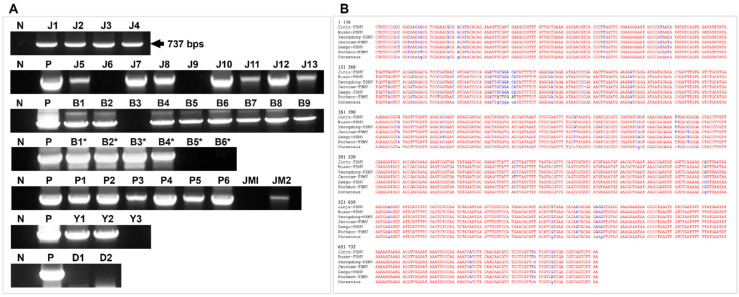
PCR analysis of collected samples for FSMV detection purpose. (**A**) PCR processing using Ash_gemini_2F/R primer was done for all collected samples from Jinju, Busan, Pocheon, Yeongdong, and Daegu. (**B**) Multiple sequence alignment of the sequences detected from all locations (one sequence from each site, i.e., J7, B6, P4, Y2, D2).

**Figure 3:**
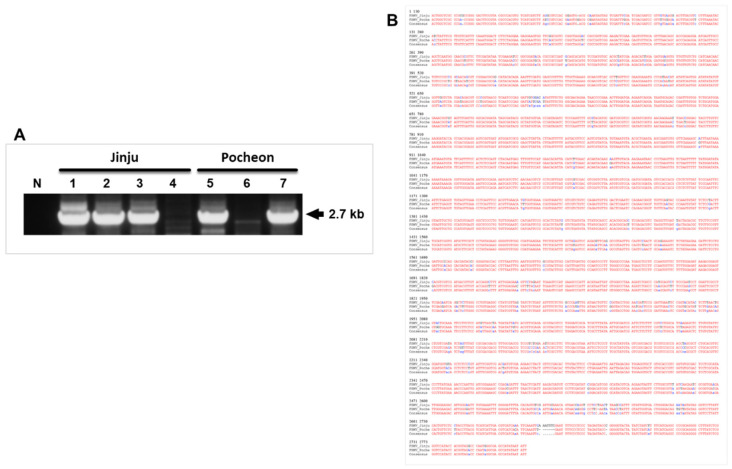
PCR and sequence analysis of full-length genome of FSMV. (**A**) Detection of full-length virus genomes from samples: J1, J2, J5, J6 from Jinju and P2, P3, P4 from Pocheon through PCR using TF2/R2 primers. (**B**) Multiple sequence alignment of the full-length sequences of FSMV detected from Jinju and Pocheon samples (Accession numbers: MZ054403 and MZ054404).

The PCR product showed a target size band of about 700 bp when subjected to gel electrophoresis, followed by sequencing (Figure 2). NCBI blasts showed 34% sequence identity to *Olea europaea geminivirus* (MW316657) and 8% to *Tomato Chino La Paz virus* (MH678590). The full-length sequences (2.7 kb) were detected from samples from all locations but at first only from Jinju and Pocheon samples and sequenced followed by GenBank submission; Accession numbers: MZ054403 and MZ054404 (Figure 3). Full-length sequences from all other locations resemble MZ054403. The sequencing results showed that it is a new virus that has not previously been reported. No satellite or DNA B could be detected in any sample using both PCR and RCA techniques (Supplementary Figure S1).

### 3.3. Genomic Features

Using the complete genomic sequences, DNA-A was investigated to identify and characterize the genomic features and the putative encoded proteins. A complete circular ssDNA virus contig was identified. In contrast to other begomoviruses, our contig comprises only DNA-A with five confirmed ORFs, while one ORF in virion-sense responsible for movement was not clear. Thorough analysis of the full genome sequence data showed an open reading frame which may encode a movement protein (V2*). All other ORFs showed the same pattern as in begomoviruses with respect to their location (Table 2). An intergenic region with a proper conserved region and stem loop structure with a nonanucleotide structure identical to that in begomoviruses was also observed. This newly identified virus lacks any satellite molecules or DNA B, which assist viruses in moving and infecting hosts.

### 3.4. Phylogenetic Relationship with Other Virus Families

**Figure 4:**
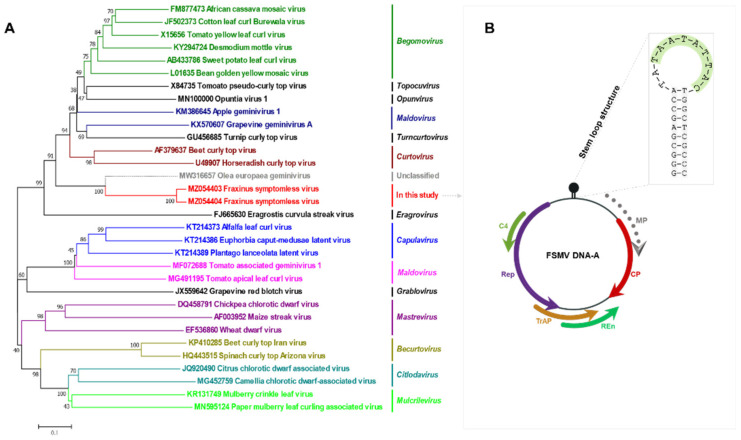
Phylogenetic relationship of FSMV detected from *F. rhynchophylla* with viruses of other genera in the family *Geminiviridae*. (**A**) Phylogenetic relationships were analyzed using the iTOL software. Nevick file for iTOL was generated using MEGA7 program. (**B**) Genomic organization of FSMV comprised of ORFs: Rep, C4, TRAP, and Ren on the complementary strand and only CP on the virion sense without MP. The stem–loop structure containing the nonanucleotide motif has been shown to the right of the phylogenetic tree.

**Figure 5:**
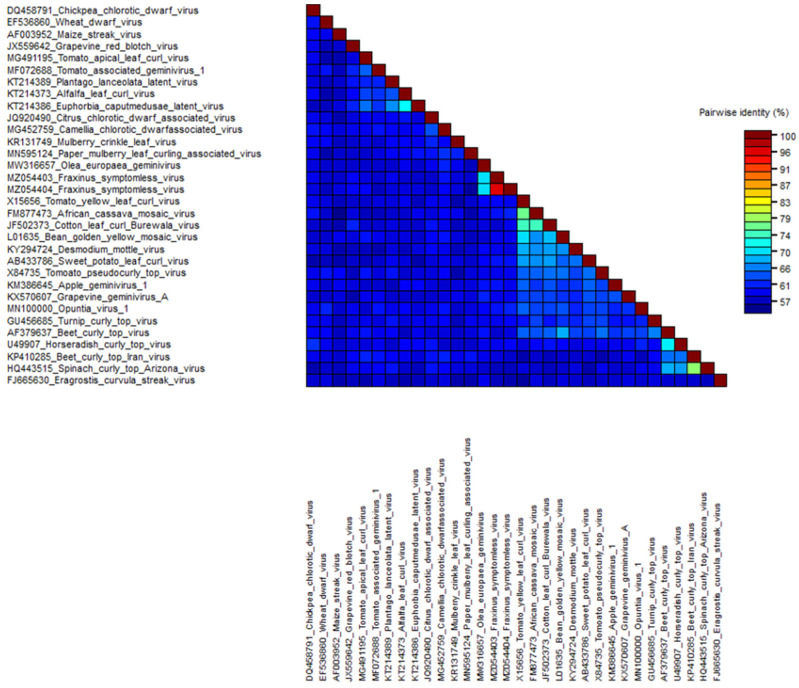
Sequence Demarcation Tool based pairwise sequence comparisons between FSMV and important members of different genera in the family *Geminiviridae*. A color-coded pairwise identity matrix was generated that processed the same sets of sequences used in phylogenetic analysis. Each colored cell represents a percentage identity score for two sequences. Colored keys indicating the correspondence between pairwise identities and the colors displayed in the matrix are presented.

The relationships between FSMV and other virus members from different genera within the family *Geminiviridae* were initially examined by comparison of the whole-genome sequences by nucleotide pairwise alignments and identity matrices. Phylogenetic analysis was run at the nucleotide level sampling representative virus sequences (Figure 4). The full-length genome sequences of these top hits were downloaded, aligned with the MUSCLE algorithm, and subjected to pairwise comparison using SDT v1.2 (Figure 5). The obtained identity matrix revealed that the sequence similarity with other viruses ranges far below the threshold for demarcation from other virus species of 91%. Neighbor-joining phylogenetic analysis of whole-genome sequences from isolates of representative species of each genus showed clusters with the unclassified *Olea europaea geminivirus* among *Becurtovirus*, *Begomovirus*, *Capulavirus*, *Citlodavirus*, *Curtovirus*, *Eragrovirus*, *Grablovirus*, *Maldovirus*, *Mastrevirus*, *Mulcrilevirus*, *Opunvirus*, *Topilevirus*, *Topocuvirus*, and *Turncurtovirus.* A comparison of most well conserved proteins such as the CP and Rep with the viruses of all 14 genera of family *Geminiviridae* was also done to show how closely related they are on a protein level (Supplementary Figure S2).

### 3.5. Southern Blotting Hybridization Analysis

To investigate whether the viral DNA was integrated into the ash tree genome, a Southern blot hybridization assay was performed. Multiple hybridization attempts were carried out using total DNA extracts from J1, B6, P4, and JM2 as probes, respectively, but none of the samples produced a noticeable specific band of the expected full-length genome size. Interestingly, DNA was clearly visualized from the CTAB extracts on the agarose gel (data not shown), but in the hybridization assay, none of them showed any specific band for the virus either as DNA or as plant genome-integrated viral sequences.

### 3.6. Strand-Specific PCR and Site-Based Detection

**Figure 6:**
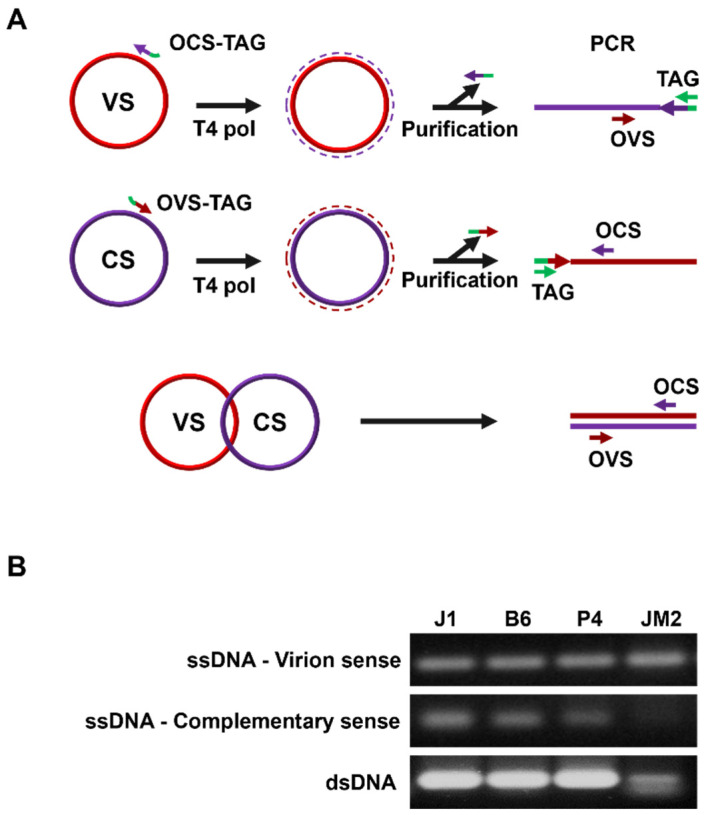
Strand-specific amplification with FSMV-infected samples. (**A**) Schematic representation of the strand-specific amplification PCR procedure for confirming the existence of virion-sense (VS) and complementary-sense (CS) DNA molecules. (**B**) Strand-specific amplification with leaf tissues of samples J1, B6, P4, and JM2 using virion-sense- and complementary-sense-specific primer sets.

Strand-specific amplification using virion-sense- and complementary-sense-specific primer sets (Figure 6A) showed that dsDNA and two ssDNA molecules (virion and complementary senses) were present in the infected samples: J1, B6, P4, and JM2, which indicates the virus replication phases in the host plants (Figure 6B).

**Figure 7:**
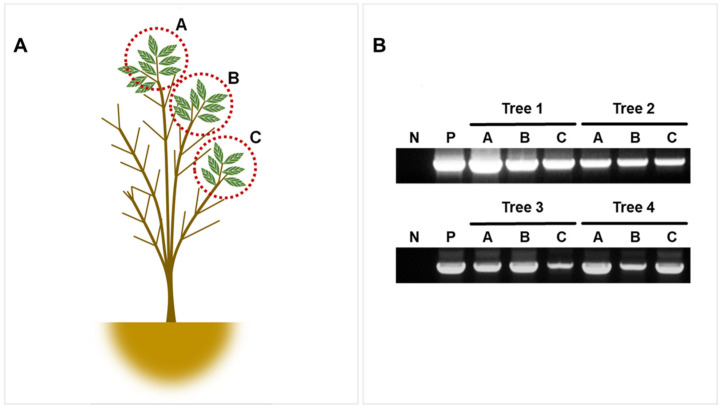
Detection of FSMV in various tree parts to confirm its infectivity. (**A**) Schematic representation of tree sampling sites, i.e., top A, middle B, and down C. (**B**) PCR processing of B1 *–B4 * ash tree samples using Ash_Gemini_2F/R primers to detect FSMV at all sampling sites.

We also checked the presence of the virus in three different locations by PCR to investigate viral replication ability and systemic movement. According to PCR, the virus was detected in four out of six samples (B1 *–B4 * were found positive and B5 *, B6 * as negative) whereas the virus was successfully detected in all three sites of positive samples, which confirmed its presence (Figure 7A,B).

### 3.7. Infectivity through Infectious Clone Inoculation

**Figure 8:**
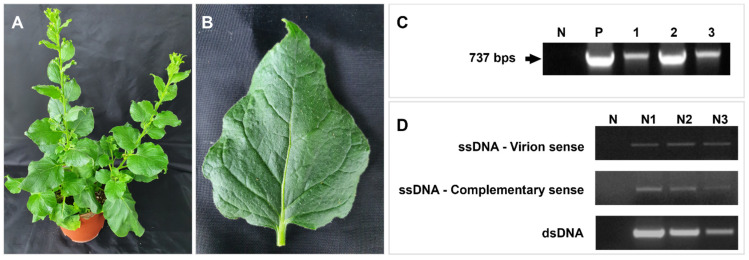
Agro-infection of FSMV in *N. benthamiana*. The results of inoculation assays for FSMV clones in (**A**) *N. benthamiana* plant and (**B**) *N. benthamiana* leaf 28 dpi. (**C**). Successful detection of FSMV using Ash_Gemini_2F/R primers in three *N. benthamiana* plants and a mock plant used as negative control in PCR processing. (**D**). Strand-specific amplification PCR procedure for confirming the existence of virion-sense (VS) and complementary-sense (CS) DNA molecules in three *N.benthamiana* plants.

*N. benthamiana* plants showed no symptoms in both mock and FSMV-inoculated groups. We could not observe any differences among all *N. benthamiana* groups inoculated. Leaf tissues were harvested and analyzed by PCR to investigate viral replication ability. According to PCR, the virus was detected, which confirmed its presence (Figure 8A,B), and viral replication was confirmed through strand-specific primers (Figure 8C). The virus replicating in *N. benthamiana* maintained the exact nucleotide sequence of the original clone (Supplementary Figure S3). PCR using vector-specific primers shows negative results which backs the virus detection on its own instead of containing the virus in different parts of the plant (Supplementary Figure S4).

## 4. Discussion

The advent of new molecular techniques (i.e., HTS technologies and RCA) has significantly broadened our knowledge of plant viruses, especially the geminiviruses. Recently, many divergent geminivirids infecting grapevine, citrus, apple, pear, *Prunus*, mulberry, chinaberry tree (*Melia azedarach*), olive tree, grey fig (*Ficus virens*), and *Jatropha multifidi* have been identified, expanding the host range to woody trees [27,28,29,44,45,47,69,70]. In this paper, we report the identification and characterization of a novel geminivirid infecting woody trees, i.e., ash trees in Korea (Figure 1, Table 1). A circular ssDNA of about 2.7 kb was identified by bioinformatic analysis in ash trees (Figure 3). Similar to other NW begomoviruses, the genome has gene encoding AC1, AC2, AC3, and AC4 proteins on the complementary strand and the coat protein (AV1) on the virion sense along with the possible AV2 protein. Unlike NW begomoviruses as well as OW begomoviruses, DNA B or satellite molecules, respectively, could not be detected in ash tree samples (Supplementary Figure S1). Despite the similar DNA-A genomic organization to NW begomoviruses, BLASTn search of the FSMV sequence revealed very low sequence identity with any begomovirus. To completely rule out the possibility of contamination of insect exudates, eggs, or larvae of insect vectors or other possible sources, PCR of four samples (J1, B6, P4, and JM2) was processed using 16S RNA primers [71] and MCOI targeting primers [72] but all had negative results (Supplementary Figure S5).

Furthermore, phylogenetic analysis showed that FSMV does not group with begomoviruses or with other approved geminiviral genera. These findings together with a neighbor-joining tree of representative full-length genomes of all genera within the *Geminiviridae* family and the overall nucleotide identity levels suggest that this virus could belong to a novel unclassified genus within the family, although information on vectors and viral particles is currently lacking (Figure 4). *F. rhynchophylla* is one of the most abundant trees found in almost all parts of Korea [1,8] and field survey revealed that the virus is widely distributed in Korea, with none of the analyzed trees infected with it showing any symptoms. FSMV was attempted to be confirmed through Southern blot hybridization assay, but this was not successful, probably because of the abundance of phenolic compounds or lower viral titer, which can inhibit the hybridization reaction or be due to a very low concentration of the virus in vivo. However, the full-length genome was detected and confirmed by using strand-specific PCR and site-based detection (Figure 6 and Figure 7).

Detection of the virus from different sites within the tree confirms the virus infection in it and its movement either from cell to cell or over long distances. Infectivity assay in *N. benthamiana* confirms the monopartite infectious nature of FSMV (Figure 8). There have been recent reports regarding the begomoviruses where movement proteins either missed or have no role [44,73]. Though the lack of a gene encoding the movement protein in the genome of any geminivirid raises many questions, proper virus movement and associated infectivity undoubtedly occur in the case of FSMV. Following this, thorough analysis of the full genome sequence data was done which showed an ORF (85–456 nt) on the virion sense which may encode a movement protein like other geminiviruses, although this would be tentative given that there is no biological data to support this yet and is a topic for further exploration.

## 5. Conclusions

FSMV, the first reported *F. rhynchophylla*-infecting DNA virus, is the latest discovered member of the family *Geminiviridae*. Despite its high divergence from other known geminiviruses, we believe that it is still a geminivirid based on its resemblance in terms of genomic structure and length. FSMV has only been found in woody trees. Two FSMV isolates, i.e., MZ054403 and MZ054404, were detected in our experiment, which share very high nucleotide sequence identities with each other. We were also able to show that cloned FSMV sequences are capable of initiating asymptomatic systemic infections in *N. benthamiana*. Although we can only confirm that FSMV is present in Korea, it remains plausible that it occurs in the Americas or other parts of the world where *Fraxinus* species are found in high numbers.

## Figures and Tables

**Table 1:** Tree ash samples collected from various locations in Korea.

No.	Location	Collection Date	Labelled as
1	Jinju	March 2019	J1
2			J2
3			J3
4			J4
5		September 2019	J5
6			J6
7			J7
8			J8
9			J9
10			J10
11			J11
12			J12
13			J13
14	Busan	October 2019	B1
15			B2
16			B3
17			B4
18			B5
19			B6
20			B7
21			B8
22			B9
23	Pocheon	September 2019	P1
24			P2
25			P3
26			P4
27			P5
28			P6
29	Jeonnam	September 2019	JM1
30			JM2
31	Yeongdong	September 2019	Y1
32			Y2
33			Y3
34	Daegu	September 2019	D1
35			D2
36	Busan	May 2020	B1 *
37			B2 *
38			B3 *
39			B4 *
40			B5 *
41			B6 *

* New Samples collected from Busan for the second time.

**Table 2:** Genomic organization and ORFs sizes of the putative *Fraxinus symptomless virus* (FSMV). The table characterizes the open reading frames of FSMV, locus, length (nt/aa), and protein identification.

#	ORF	Locus	nt/aa	Strand	Protein
1	V1	153–905	753/251	Positive	Coat Protein
2	V2*	85–416	372/123	Positive	MP
3	C3	902–1342	441/147	Negative	REn
4	C2	1029–1487	459/153	Negative	TrAP
5	C1	1399–2481	1083/361	Negative	Rep
6	C4	2255–2416	162/54	Negative	C4

* V2 is the possible movement protein found in the genomic organization of FSMV.

## Data Availability

FSMV genomic sequences have been submitted in GenBank under accession numbers MZ054403 and MZ054404.

## Supplementary Materials

The following are available online at https://www.mdpi.com/article/10.3390/v13122385/s1. Supplementary Figure S1. PCR processing to detect FSMV associated molecules. (A) Detection of DNA B, (B) detection of alpha-satellite molecule, and (C) detection of beta -satellite molecules. Supplementary Figure S2. Comparison of FSMV with all members of the genera in family *Geminiviridae* on protein level. (A) Construction of phylogenetic tree, (B) Sequence Demarcation Tool (SDT) based pairwise sequence comparisons, and (C) identity scores in a matrix based upon coat protein, whereas (D) the construction of phylogenetic tree, (E) SDT-based pairwise sequence comparisons, and (F) identity scores in the matrix based upon Rep protein. Supplementary Figure S3. Multiple sequence alignment of FSMV extracted from three *N. benthamiana* plants (N1, N2, and N3) used in infectivity assay. Supplementary Figure S4. Detection of pCAMBIA1303 plasmid containing the virus using vector specific primers. (A) Gel electrophoresis of PCR product obtained using vector specific primers. N: Negative control with mock plant extracted DNA as template, P: Positive control with pCAMBIA1303 plasmid as template, N1-N3: *N. benthamiana* plants used in infectivity assay. (B) Details of vector-specific primers (pCam-F/R) used for amplification purpose. Supplementary Figure S5. PCR processing to check the possibility of contamination from insect exudates, eggs, or larvae of insect vectors. (A) PCR processing of four ash tree samples, i.e., J1, B6, P4, and JM2 using 16SRNA primers and MCOI targeting primers, respectively. (B) Details of 16SRNA primers and MCOI targeting primers used for amplification purpose. Supplementary Figure S6. Full length sequences of detected FSMV isolates. (A) Sequence and genetic map of MZ054403 and (B) the sequence and genetic map of MZ054404, respectively. Supplementary Table S1. Primers used to detect FSMV DNA-A, DNA-B and to confirm the existence of virion-sense (VS) and complementary-sense (CS) DNA molecules in strand-specific amplification PCR procedure.

## References

[1] Sarfraz I., Rasul A., Jabeen F., Younis T., Zahoor M.K., Arshad M., Ali M. (2017). *Fraxinus*: A Plant with Versatile Pharmacological and Biological Activities. eCAM.

[2] Younis T., Khan M.R., Sajid M., Majid M., Zahra Z., Shah N.A. (2016). *Fraxinus xanthoxyloides* leaves reduced the level of inflammatory mediators during in vitro and in vivo studies. BMC Complement Altern. Med..

[3] Fernández-Manjarrés J., Gérard P., Dufour J., Raquin C. (2006). Differential patterns of morphological and molecular hybridization between *Fraxinus excelsior* L. and *Fraxinus angustifolia* Vahl (Oleaceae) in east ern and western France. Mol. Ecol..

[4] Hinsinger D.D., Gaudeul M., Couloux A., Bousquet J., Frascaria-Lacoste N. (2014). The phylogeography of Eurasian *Fraxinus* species reveals ancient transcontinental reticulation. Mol. Phylogenet. Evol..

[5] Kostova I., Iossifova T. (2007). Chemical components of *Fraxinus* species. Fitoterapia.

[6] Wu Z.-B., Liu Y., Tian S.-S., Wen C. (2014). Chemical constituents of the stem bark of *Fraxinus rhynchophylla*. Chem. Nat. Compd..

[7] Torres M., Palomares O., Quiralte J., Pauli G., Rodríguez R., Villalba M. (2015). An Enzymatically active β-1, 3-Glucanase from ash pollen with allergenic properties: A particular member in the Oleaceae Family. PLoS ONE.

[8] Jung B.-N., Choi Y.-J., Shin H.-D., Park J.-H. (2020). Macruropyxis fraxini on *Fraxinus rhynchophylla*: Confirmation in the Korean Peninsula after 82 Years and the First Record in South Korea. Mycobiology.

[9] Luo Y., Cobb R.E., Zhao H. (2014). Recent advances in natural product discovery. Curr. Opin. Biotechnol..

[10] Kassanis B., Macfarlane I. (1964). Transmission of Tobacco Necrosis Virus to Tobacco Callus Tissues by Zoospores of *Olpidium brassicae*. Nature.

[11] Reichmann M. (1964). The satellite tobacco necrosis virus: A single protein and its genetic code. Proc. Natl. Acad. Sci. USA.

[12] Schneider I. (1971). Characteristics of a satellite-like virus of tobacco ringspot virus. Virol. J..

[13] Hollings M., Stone O., Dale W. (1972). Tomato ringspot virus in Pelargonium in England. Plant Pathol..

[14] Thomas W., Procter C. (1972). Arabis mosaic virus in *Cyphomandra betaceae* Sendt. N. Z. J. Agric. Res..

[15] Wilson T.M.A. (1984). Cotranslational disassembly of tobacco mosaic virus in vitro. Virol. J..

[16] Kay L.E.W.M. (1986). Stanley’s crystallization of the tobacco mosaic virus, 1930–1940. Isis Int. Rev. Devoted Hist. Sci. Its Cult. Influ..

[17] Harrison B.D., Murant A.F., Harrison B.D., Murant A.F. (1996). Nepoviruses: Ecology and Control. The Plant Viruses: Polyhedral Virions and Bipartite RNA Genomes.

[18] Quadt-Hallmann A., Löw A., Hamacher J. (1996). Distribution of cherry leaf roll nepovirus (CLRV) in leaves of deciduous forest trees and herbaceous plants detected by tissue print immunopressblotting (TPI) of whole leaf blades/Verteilung des Kirschenblattroll-Nepovirus (CLRV) in Blättern von Forstgehölzen und krautigen Pflanzen. Virusnachweis an ganzen Blattspreiten mit dem Immungewebepressabdruckverfahren. J. Plant Dis. Prot..

[19] Fillhart R.C., Bachand G.D., Castello J.D. (1998). Detection of Infectious *Tobamoviruses* in Forest Soils. Appl. Environ. Microbiol..

[20] Villamor D.E.V., Ho T., Al Rwahnih M., Martin R.R., Tzanetakis I.E. (2019). High Throughput Sequencing For Plant Virus Detection and Discovery. Phytopathology.

[21] Roossinck M.J., Martin D.P., Roumagnac P. (2015). Plant Virus Metagenomics: Advances in Virus Discovery. Phytopathology.

[22] Rodríguez-Negrete E.A., Morales-Aguilar J.J., Domínguez-Duran G., Torres-Devora G., Camacho-Beltrán E., Leyva-López N.E., Voloudakis A.E., Bejarano E.R., Méndez-Lozano J. (2019). High-Throughput Sequencing Reveals Differential *Begomovirus* Species Diversity in Non-Cultivated Plants in Northern-Pacific Mexico. Viruses.

[23] Fontenele R.S., Salywon A.M., Majure L.C., Cobb I.N., Bhaskara A., Avalos-Calleros J.A., Argüello-Astorga G.R., Schmidlin K., Khalifeh A., Smith K. (2020). A Novel Divergent *Geminivirus* Identified in Asymptomatic New World *Cactaceae* Plants. Viruses.

[24] Zerbini F.M., Briddon R.W., Idris A., Martin D.P., Moriones E., Navas-Castillo J., Rivera-Bustamante R., Roumagnac P., Varsani A., Consortium I.R. (2017). ICTV Virus Taxonomy Profile: *Geminiviridae*. J. Gen. Virol..

[25] Varsani A., Roumagnac P., Fuchs M., Navas-Castillo J., Moriones E., Idris A., Briddon R.W., Rivera-Bustamante R., Murilo Zerbini F., Martin D.P. (2017). Capulavirus and Grablovirus: Two new genera in the family *Geminiviridae*. Arch. Virol..

[26] Perry K.L., McLane H., Thompson J.R., Fuchs M. (2018). A novel grablovirus from non-cultivated grapevine (*Vitis* sp.) in North America. Arch. Virol..

[27] Loconsole G., Saldarelli P., Doddapaneni H., Savino V., Martelli G.P., Saponari M. (2012). Identification of a single-stranded DNA virus associated with citrus chlorotic dwarf disease, a new member in the family *Geminiviridae*. Virol. J..

[28] Ma Y., Navarro B., Zhang Z., Lu M., Zhou X., Chi S., Di Serio F., Li S. (2015). Identification and molecular characterization of a novel monopartite *geminivirus* associated with mulberry mosaic dwarf disease. J. Gen. Virol..

[29] Liang P., Navarro B., Zhang Z., Wang H., Lu M., Xiao H., Wu Q., Zhou X., Di Serio F., Li S. (2015). Identification and characterization of a novel *geminivirus* with a monopartite genome infecting apple trees. J. Gen. Virol..

[30] Zhang S., Shen P., Li M., Tian X., Zhou C., Cao M. (2018). Discovery of a novel *geminivirus* associated with camellia chlorotic dwarf disease. Arch. Virol..

[31] Vaghi Medina C.G., Teppa E., Bornancini V.A., Flores C.R., Marino-Buslje C., López Lambertini P.M. (2018). Tomato Apical Leaf Curl Virus: A Novel, Monopartite *Geminivirus* Detected in Tomatoes in Argentina. Front. Microbiol..

[32] Fontenele R.S., Abreu R.A., Lamas N.S., Alves-Freitas D.M.T., Vidal A.H., Poppiel R.R., Melo F.L., Lacorte C., Martin D.P., Campos M.A. (2018). Passion Fruit Chlorotic Mottle Virus: Molecular Characterization of a New Divergent *Geminivirus* in Brazil. Viruses.

[33] Fontenele R.S., Lamas N.S., Lacorte C., Lacerda A.L.M., Varsani A., Ribeiro S.G. (2017). A novel *geminivirus* identified in tomato and cleome plants sampled in Brazil. Virus Res..

[34] Zhang R., Wu X., Jiang X., Wu X., Luan X., Cheng X. (2020). Molecular characterization of common bean curly stunt virus: A novel recombinant *geminivirus* in China. Arch. Virol..

[35] Mansoor S., Briddon R.W., Zafar Y., Stanley J. (2003). *Geminivirus* disease complexes: An emerging threat. Trends Plant Sci..

[36] Jeske H., de Villiers E.-M., Hausen H.Z. (2009). Geminiviruses. TT Viruses: The Still Elusive Human Pathogens.

[37] Lazarowitz S.G., Shepherd R.J. (1992). *Gemi*-*iviruses*: Genome structure and gene function. Crit. Rev. Plant Sci.

[38] Hanley-Bowdoin L., Settlage S.B., Orozco B.M., Nagar S., Robertson D. (1999). *Geminiviruses*: Models for Plant DNA Replication, Transcription, and Cell Cycle Regulation. Crit. Rev. Plant Sci..

[39] Hehnle S., Wege C., Jeske H. (2004). Interaction of DNA with the movement proteins of *geminiviruses* revisited. Virol. J..

[40] Rojas M.R., Macedo M.A., Maliano M.R., Soto-Aguilar M., Souza J.O., Briddon R.W., Kenyon L., Bustamante R.F.R., Zerbini F.M., Adkins S. (2018). World Management of *Geminiviruses*. Annu. Rev. Phytopathol..

[41] Moffat A.S. (1999). *Geminiviruses* Emerge as Serious Crop Threat. Science.

[42] Lal A., Vo T.T.B., Sanjaya I.G.N.P.W., Ho P.T., Kim J.-K., Kil E.-J., Lee S. (2020). *Nanovirus* Disease Complexes: An Emerging Threat in the Modern Era. Front. Plant Sci..

[43] Kraberger S., Geering A.D.W., Walters M., Martin D.P., Varsani A. (2017). Novel *mastreviruses* identified in Australian wild rice. Virus Res..

[44] Chiumenti M., Greco C., De Stradis A., Loconsole G., Cavalieri V., Altamura G., Zicca S., Saldarelli P., Saponari M. (2021). Olea Europaea *Geminivirus*: A Novel Bipartite *Geminivirid* Infecting Olive Trees. Viruses.

[45] Lal A., Kil E.-J., Vo T.T.B., Fadhila C., Ho P.T., Shuja M.N., Ali M., Lee S. (2020). First Report of *Duranta* leaf curl virus Infecting *Ficus virens* Showing Leaf Curl Symptoms in Pakistan. Plant Dis..

[46] Lal A., Kil E.-J., Rauf K., Ali M., Lee S. (2020). First Report of Papaya leaf curl virus Associated with Leaf Curl Disease in Cestrum nocturnum in Pakistan. Plant Dis..

[47] Jones R.A.C., Coutts B.A. (2015). Spread of introduced viruses to new plants in natural ecosystems and the threat this poses to plant biodiversity. Mol. Plant Pathol..

[48] Alexander H.M., Mauck K.E., Whitfield A.E., Garrett K.A., Malmstrom C.M. (2014). Plant-virus interactions and the agro-ecological interface. Eur. J. Plant Pathol..

[49] Elena S.F., Fraile A., García-Arenal F., Maramorosch K., Murphy F.A. (2014). Evolution and Emergence of Plant Viruses. Advance Virus Research.

[50] Jones R.A.C. (2009). Plant virus emergence and evolution: Origins, new encounter scenarios, factors driving emergence, effects of changing world conditions, and prospects for control. Virus Res..

[51] Shepherd L.D., McLay T.G. (2011). Two micro-scale protocols for the isolation of DNA from polysaccharide-rich plant tissue. J. Plant Res..

[52] Shepherd D.N., Martin D.P., Lefeuvre P., Monjane A.L., Owor B.E., Rybicki E.P., Varsani A. (2008). A protocol for the rapid isolation of full *geminivirus* genomes from dried plant tissue. J. Virol. Methods.

[53] Bankevich A., Nurk S., Antipov D., Gurevich A.A., Dvorkin M., Kulikov A.S., Lesin V.M., Nikolenko S.I., Pham S., Prjibelski A.D. (2012). SPAdes: A new genome assembly algorithm and its applications to single-cell sequencing. J. Comput. Biol..

[54] Altschul S.F., Madden T.L., Schäffer A.A., Zhang J., Zhang Z., Miller W., Lipman D.J. (1997). Gapped BLAST and PSI-BLAST: A new generation of protein database search programs. Nucleic Acids Res..

[55] O’Leary N.A., Wright M.W., Brister J.R., Ciufo S., Haddad D., McVeigh R., Rajput B., Robbertse B., Smith-White B., Ako-Adjei D. (2016). Reference sequence (RefSeq) database at NCBI: Current status, taxonomic expansion, and functional annotation. Nucleic Acids Res..

[56] Briddon R., Bull S., Mansoor S., Amin I., Markham P. (2002). Universal primers for the PCR-mediated amplification of DNA β *Mol*. Biotechnol..

[57] Bull S., Briddon R., Markham P. (2003). Universal primers for the PCR-mediated amplification of DNA 1: A satellite-like molecule associated with *begomovirus*-DNA β complexes. Mol. Biotechnol..

[58] Rojas M., Gilbertson R., Maxwell D. (1993). Use of degenerate primers in the polymerase chain reaction to detect whitefly-transmitted *geminiviruses*. Plant Dis..

[59] Corpet F. (1988). Multiple sequence alignment with hierarchical clustering. Nucleic Acids Res..

[60] Higo K., Ugawa Y., Iwamoto M., Korenaga T. (1999). Plant cis-acting regulatory DNA elements (PLACE) database: 1999. Nucleic Acids Res..

[61] Muhire B.M., Varsani A., Martin D.P. (2014). SDT: A Virus Classification Tool Based on Pairwise Sequence Alignment and Identity Calculation. PLoS ONE.

[62] Kumar S., Stecher G., Tamura K. (2016). Evolution, MEGA7: Molecular evolutionary genetics analysis version 7.0 for bigger datasets. Mol. Biol. Evol..

[63] Walker P.J., Siddell S.G., Lefkowitz E.J., Mushegian A.R., Adriaenssens E.M., Dempsey D.M., Dutilh B.E., Harrach B., Harrison R.L., Hendrickson R.C. (2020). Changes to virus taxonomy and the Statutes ratified by the International Committee on Taxonomy of Viruses (2020). Arch. Virol..

[64] Southern E.M. (1975). Detection of specific sequences among DNA fragments separated by gel electrophoresis. J. Mol. Biol..

[65] Kil E.-J., Kim S., Lee Y.-J., Byun H.-S., Park J., Seo H., Kim C.-S., Shim J.-K., Lee J.-H., Kim J.-K. (2016). Tomato yellow leaf curl virus (TYLCV-IL): A seed-transmissible geminivirus in tomatoes. Sci. Rep..

[66] Rodríguez-Negrete E.A., Sánchez-Campos S., Cañizares M.C., Navas-Castillo J., Moriones E., Bejarano E.R., Grande-Pérez A. (2014). A sensitive method for the quantification of virion-sense and complementary-sense DNA strands of circular single-stranded DNA viruses. Sci. Rep..

[67] Kil E.-J., Park J., Choi E.-Y., Byun H.-S., Lee K.-Y., An C.G., Lee J.-H., Lee G.-S., Choi H.-S., Kim C.-S. (2018). Seed transmission of Tomato yellow leaf curl virus in sweet pepper (*Capsicum annuum*). Eur. J. Plant Pathol..

[68] Seol E., Jung Y., Lee J., Cho C., Kim T., Rhee Y., Lee S. (2008). In planta transformation of Notocactus scopa cv. Soonjung by Agrobacterium tumefaciens. Plant Cell Rep..

[69] Al Rwahnih M., Alabi O.J., Westrick N.M., Golino D. (2018). Prunus *geminivirus* A: A Novel *Grablovirus* Infecting *Prunus* spp.. Plant Dis..

[70] Al Rwahnih M., Alabi O.J., Westrick N.M., Golino D., Rowhani A. (2017). Description of a Novel Monopartite *Geminivirus* and Its Defective Subviral Genome in Grapevine. Phytopathology.

[71] Simon C., Frati F., Beckenbach A., Crespi B., Liu H., Flook P. (1994). Evolution, Weighting, and Phylogenetic Utility of Mitochondrial Gene Sequences and a Compilation of Conserved Polymerase Chain Reaction Primers. Ann. Entomol. Soc. Am..

[72] Wang J.-F., Jiang L.-Y., Qiao G.-X. (2011). Use of a mitochondrial COI sequence to identify species of the subtribe Aphidina (Hemiptera, Aphididae). Zookeys.

[73] Hak H., Levy Y., Chandran S.A., Belausov E., Loyter A., Lapidot M., Gafni Y. (2015). TYLCV-Is movement in planta does not require V2 protein. Virol. J..

