# Supplementary material for: Identification of a Novel Geminivirus in Fraxinus rhynchophylla in Korea
Source: Viruses. 2021 Nov 28;13(12):2385. doi: 10.3390/v13122385 (PMC8705360; doi:10.3390/v13122385)
Supplement: Supplementary file 1 [file viruses-13-02385-s001.zip › viruses-1426485-supplementary.pdf]

Supplementary Data

Supplementary Figure S1

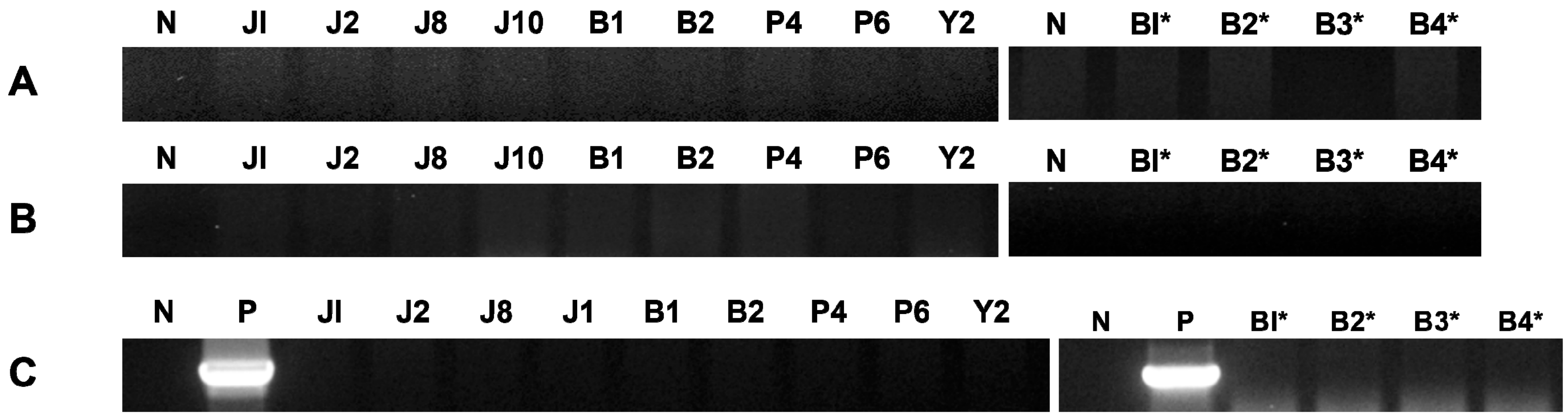

Supplementary Figure S2 Coat Protein

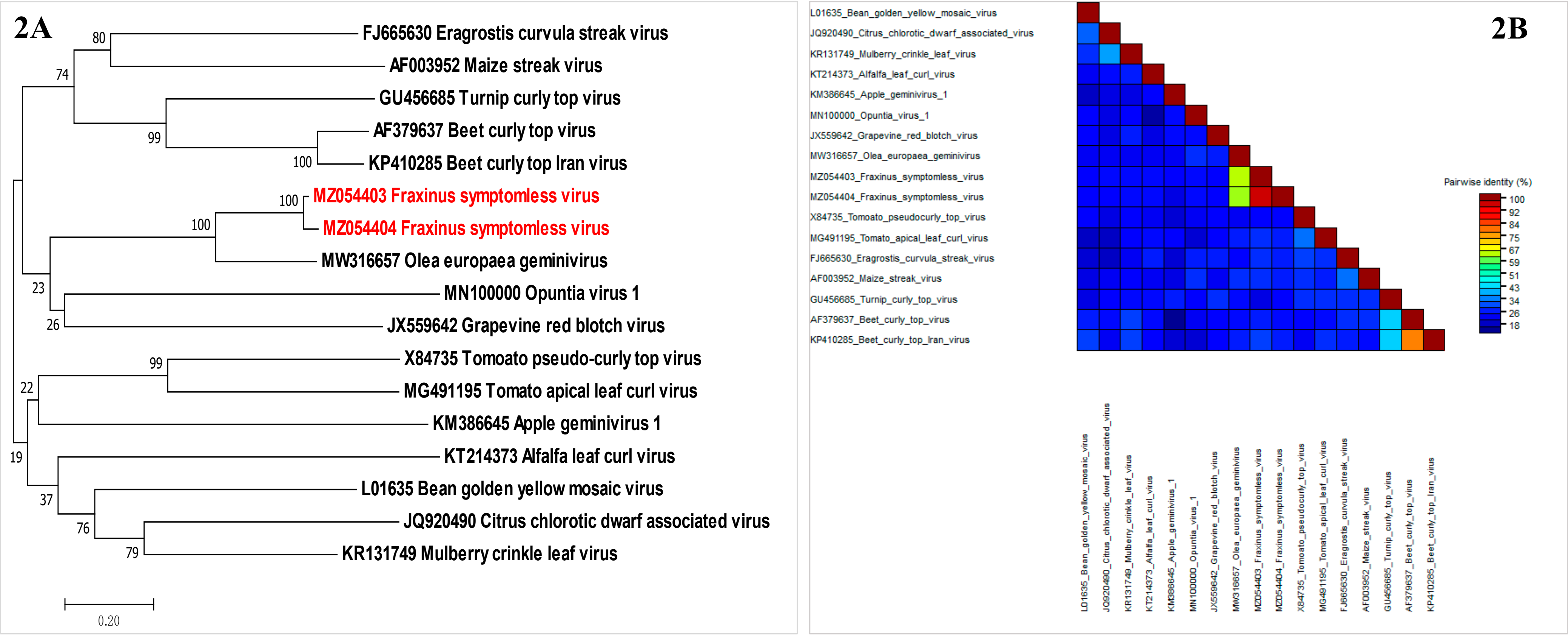

2C

CP_Identity scores

>L01635_Bean_golden_yellow_mosaic_virus	100.001
>JQ920490_Citrus_chlorotic_dwarf_associated_virus	31.601,100.001
>KR131749_Mulberry_crinkle_leaf_virus	28.001,37.601,100.001
>KT214373_Alfalfa_leaf_curl_virus	21.801,24.801,26.001,100.001
>KM386645_Apple_geminivirus_1	18.001,20.801,20.801,23.301,100.001
>MN100000_Opuntia_virus_1	22.901,21.201,23.301,14.601,24.901,100.001
>JX559642_Grapevine_red_blotch_virus	22.901,20.601,26.101,21.101,24.501,25.001,100.001
>MW316657_Olea_europaea_geminivirus	21.801,22.501,22.101,22.301,21.801,27.801,25.601,100.001
>MZ054403_Fraxinus_symptomless_virus	23.001,24.301,24.401,25.001,23.701,25.101,23.501,63.601,100.001
>MZ054404_Fraxinus_symptomless_virus	23.601,23.901,24.901,25.001,21.201,24.701,23.501,62.401,95.601,100.001
>X84735_Tomoato_pseudocurly_top_virus	24.001,21.301,22.801,22.501,19.301,21.601,23.301,22.401,23.201,23.301,100.001
>MG491195_Tomato_apical_leaf_curl_virus	18.301,18.601,23.501,24.701,22.801,19.801,23.201,26.301,27.201,25.901,32.801,100.001
>FJ665630_Eragrostis_curvula_streak_virus	19.701,17.401,21.401,25.701,19.301,25.801,25.901,23.901,26.901,26.101,28.401,27.601,100.001
>AF003952_Maize_streak_virus	20.201,21.601,22.901,21.801,20.501,27.001,22.101,27.601,27.001,26.601,27.001,26.501,33.601,100.001
>GU456685_Turnip_curly_top_virus	20.301,23.801,24.201,24.701,25.501,24.101,27.001,24.401,20.901,23.001,28.101,28.201,26.901,27.501,100.001
>AF379637_Beet_curly_top_virus	26.701,24.301,29.601,24.901,13.901,22.301,23.301,25.301,26.301,25.301,23.001,25.101,27.301,28.201,41.401,100.001
>KP410285_Beet_curly_top_Iran_virus	28.601,22.301,28.701,23.601,18.801,19.301,23.701,26.301,28.301,25.501,22.601,27.401,25.801,25.301,41.101,78.501,100.001

Rep Protein

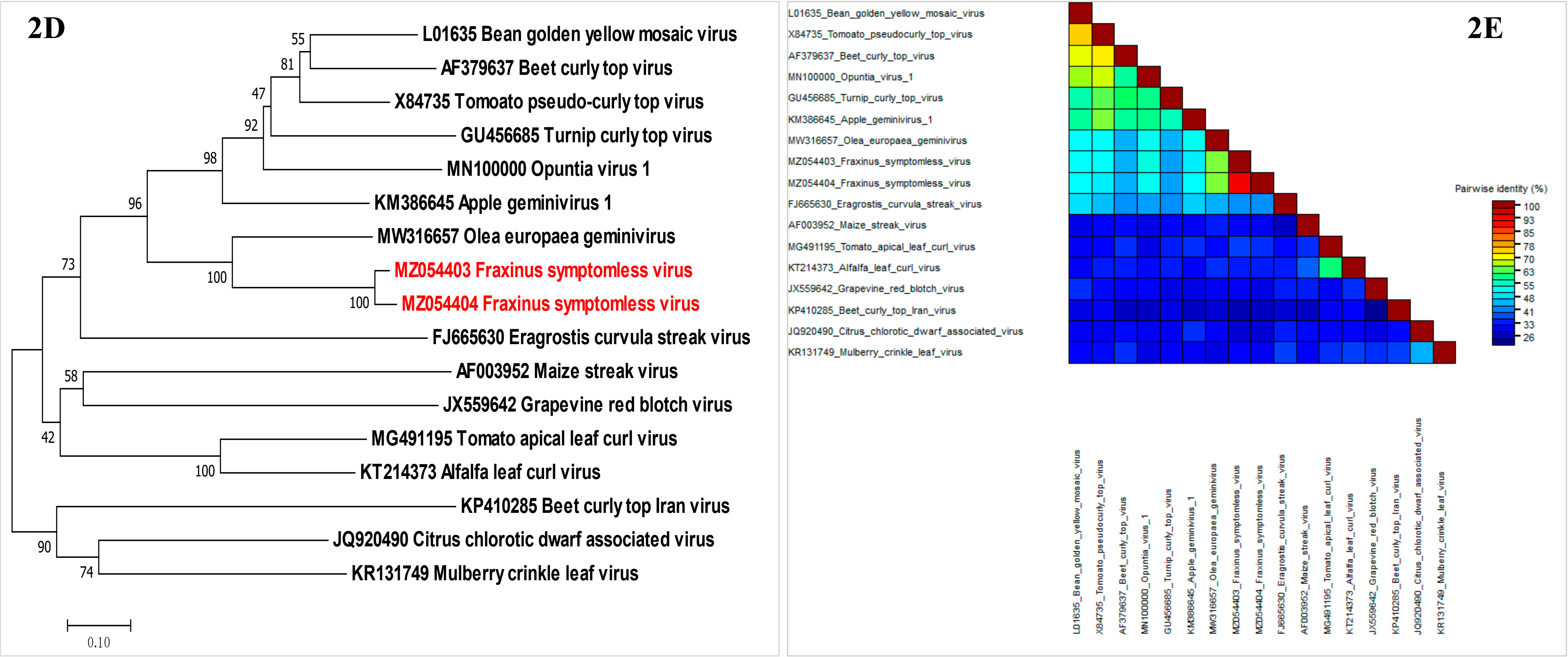

2D
L01635 Bean golden yellow mosaic virus
AF379637 Beet curly top virus
X84735 Tomoato pseudo-curly top virus
GU456685 Turnip curly top virus
MN100000 Opuntia virus 1
KM386645 Apple geminivirus 1
MW316657 Olea europaea geminivirus
MZ054403 Fraxinus symptomless virus
MZ054404 Fraxinus symptomless virus
FJ665630 Eragrostis curvula streak virus
AF003952 Maize streak virus
JX559642 Grapevine red blotch virus
MG491195 Tomato apical leaf curl virus
KT214373 Alfalfa leaf curl virus
KP410285 Beet curly top Iran virus
JQ920490 Citrus chlorotic dwarf associated virus
KR131749 Mulberry crinkle leaf virus
55
81
47
92
98
96
73
100
100
58
42
100
90
74
0.10
2E
L01635_Bean_golden_yellow_mosaic_virus
X84735_Tomoato_pseudocurly_top_virus
AF379637_Beet_curly_top_virus
MN100000_Opuntia_virus_1
GU456685_Turnip_curly_top_virus
KM386645_Apple_geminivirus_1
MW316657_Olea_europaea_geminivirus
MZ054403_Fraxinus_symptomless_virus
MZ054404_Fraxinus_symptomless_virus
FJ665630_Eragrostis_curvula_streak_virus
AF003952_Maize_streak_virus
MG491195_Tomato_apical_leaf_curl_virus
KT214373_Alfalfa_leaf_curl_virus
JX559642_Grapevine_red_blotch_virus
KP410285_Beet_curly_top_Iran_virus
JQ920490_Citrus_chlorotic_dwarf_associated_virus
KR131749_Mulberry_crinkle_leaf_virus
Pairwise identity (%)
100
93
85
78
70
63
55
48
41
33
26

2F

Rep_Identity scores

>L01635_Bean_golden_yellow_mosaic_virus	100.001
>X84735_Tomoato_pseudocurly_top_virus_Rep	73.601,100.001
>AF379637_Beet_curly_top_virus	68.801,71.001,100.001
>MN100000_Opuntia_virus_1	65.601,67.401,58.201,100.001
>GU456685_Turnip_curly_top_virus	56.801,62.401,61.901,59.301,100.001
>KM386645_Apple_geminivirus_1	58.201,63.401,57.301,58.601,54.701,100.001
>MW316657_Olea_europaea_geminivirus	50.101,49.901,44.401,51.901,44.101,51.101,100.001
>MZ054403_Fraxinus_symptomless_virus	50.601,50.601,44.201,52.401,42.901,49.401,63.901,100.001
>MZ054404_Fraxinus_symptomless_virus	50.601,49.401,43.901,52.101,43.101,50.001,64.401,94.201,100.001
>FJ665630_Eragrostis_curvula_streak_virus	47.301,45.201,41.901,42.401,42.101,46.001,43.901,42.101,41.501,100.001
>AF003952_Maize_streak_virus	28.201,30.701,31.501,28.301,30.401,29.801,33.201,29.001,29.001,25.501,100.001
>MG491195_Tomato_apical_leaf_curl_virus	29.701,30.301,33.601,27.601,33.101,34.001,31.601,36.001,34.801,31.301,33.901,100.001
>KT214373_Alfalfa_leaf_curl_virus	28.801,32.601,32.801,31.701,33.301,31.001,34.201,32.601,32.901,32.701,37.801,59.801,100.001
>JX559642_Grapevine_red_blotch_virus	34.501,31.701,30.801,30.501,27.401,27.901,29.301,29.201,28.501,33.201,33.501,29.801,34.001,100.001
>KP410285_Beet_curly_top_Iran_virus	27.501,29.201,25.001,26.101,28.101,25.401,26.301,24.601,26.001,26.301,26.101,28.901,30.601,21.801,100.001
>JQ920490_Citrus_chlorotic_dwarf_associated_virus	29.901,29.401,31.501,29.501,28.801,33.801,30.301,26.901,26.901,32.401,31.601,30.601,31.101,28.401,31.301,100.001
>KR131749_Mulberry_crinkle_leaf_virus	30.901,31.701,33.801,28.001,30.501,29.601,27.401,30.501,28.901,35.101,31.001,33.901,35.301,33.701,36.001,44.701,100.001

Supplementary Figure S3

```
1 130
MZ054403     CCACGTGTCA TCATCTTAGG CGTCCACGGA GTGACGCAAA TAGTAGTCGA TTTCGATCGA CGATCCGTTG TGAAGGACTT TACGTCCTTT AAATACATCT ATTTCGTTGT TCATTTCAAA TGGACTCTTC
N1           CCACGTGTCA TCATCTTAGG CGTCCACGGA GTGACGCAAA TAGTAGTCGA TTTCGATCGA CGATCCGTTG TGAAGGACTT TACGTCCTTT AAATACATCT ATTTCGTTGT TCATTTCAAA TGGACTCTTC
N3           CCACGTGTCA TCATCTTAGG CGTCCACGGA GTGACGCAAA TAGTAGTCGA TTTCGATCGA CGATCCGTTG TGAAGGACTT TACGTCCTTT AAATACATCT ATTTCGTTGT TCATTTCAAA TGGACTCTTC
N2           CCACGTGTCA TCATCTTAGG CGTCCACGGA GTGACGCAAA TAGTAGTCGA TTTCGATCGA CGATCCGTTG TGAAGGACTT TACGTCCTTT AAATACATCT ATTTCGTTGT TCATTTCAAA TGGACTCTTC
Consensus    CCACGTGTCA TCATCTTAGG CGTCCACGGA GTGACGCAAA TAGTAGTCGA TTTCGATCGA CGATCCGTTG TGAAGGACTT TACGTCCTTT AAATACATCT ATTTCGTTGT TCATTTCAAA TGGACTCTTC

131 260
MZ054403     TAGGAAGAGG AAGTGGTTCG GCGGTCCGGC TAGGACCGCC GGTCGGAAGA CTCGAAGAGT GTTGCAGTTT AACAGCACCC AGAGGAATCA GTTGCCAGCT CAATGGCAAC GCGTTCTTCG ATATAATCGA
N1           TAGGAAGAGG AAGTGGTTCG GCGGTCCGGC TAGGACCGCC GGTCGGAAGA CTCGAAGAGT GTTGCAGTTT AACAGCACCC AGAGGAATCA GTTGCCAGCT CAATGGCAAC GCGTTCTTCG ATATAATCGA
N3           TAGGAAGAGG AAGTGGTTCG GCGGTCCGGC TAGGACCGCC GGTCGGAAGA CTCGAAGAGT GTTGCAGTTT AACAGCACCC AGAGGAATCA GTTGCCAGCT CAATGGCAAC GCGTTCTTCG ATATAATCGA
N2           TAGGAAGAGG AAGTGGTTCG GCGGTCCGGC TAGGACCGCC GGTCGGAAGA CTCGAAGAGT GTTGCAGTTT AACAGCACCC AGAGGAATCA GTTGCCAGCT CAATGGCAAC GCGTTCTTCG ATATAATCGA
Consensus    TAGGAAGAGG AAGTGGTTCG GCGGTCCGGC TAGGACCGCC GGTCGGAAGA CTCGAAGAGT GTTGCAGTTT AACAGCACCC AGAGGAATCA GTTGCCAGCT CAATGGCAAC GCGTTCTTCG ATATAATCGA

261 390
MZ054403     AGATCCGGCG GATACACGCC GCCGATGCAG CACATGTCCG ATGTGCACGC TATCGAAGAC ATTGGAGATG GAGGTGTTGT TGTCTGCATC AACAACTGTC CCGCCGGCAA CAGCGTCGGA ACGCGGCATA
N1           AGATCCGGCG GATACACGCC GCCGATGCAG CACATGTCCG ATGTGCACGC TATCGAAGAC ATTGGAGATG GAGGTGTTGT TGTCTGCATC AACAACTGTC CCGCCGGCAA CAGCGTCGGA ACGCGGCATA
N3           AGATCCGGCG GATACACGCC GCCGATGCAG CACATGTCCG ATGTGCACGC TATCGAAGAC ATTGGAGATG GAGGTGTTGT TGTCTGCATC AACAACTGTC CCGCCGGCAA CAGCGTCGGA ACGCGGCATA
N2           AGATCCGGCG GATACACGCC GCCGATGCAG CACATGTCCG ATGTGCACGC TATCGAAGAC ATTGGAGATG GAGGTGTTGT TGTCTGCATC AACAACTGTC CCGCCGGCAA CAGCGTCGGA ACGCGGCATA
Consensus    AGATCCGGCG GATACACGCC GCCGATGCAG CACATGTCCG ATGTGCACGC TATCGAAGAC ATTGGAGATG GAGGTGTTGT TGTCTGCATC AACAACTGTC CCGCCGGCAA CAGCGTCGGA ACGCGGCATA

391 520
MZ054403     CACAGAAAGT TCGATGGAGC CGTTTGTTGC TGAAAGGCAA CGTCACCCTT GGTTCCGAAG GAAATGCCCA TAACATATAT CAGTGGATAT ATATGTGGTT GGTCTACGAC AGACGTCCCG GTAACGTCAA
N1           CACAGAAAGT TCGATGGAGC CGTTTGTTGC TGAAAGGCAA CGTCACCCTT GGTTCCGAAG GAAATGCCCA TAACATATAT CAGTGGATAT ATATGTGGTT GGTCTACGAC AGACGTCCCG GTAACGTCAA
N3           CACAGAAAGT TCGATGGAGC CGTTTGTTGC TGAAAGGCAA CGTCACCCTT GGTTCCGAAG GAAATGCCCA TAACATATAT CAGTGGATAT ATATGTGGTT GGTCTACGAC AGACGTCCCG GTAACGTCAA
N2           CACAGAAAGT TCGATGGAGC CGTTTGTTGC TGAAAGGCAA CGTCACCCTT GGTTCCGAAG GAAATGCCCA TAACATATAT CAGTGGATAT ATATGTGGTT GGTCTACGAC AGACGTCCCG GTAACGTCAA
Consensus    CACAGAAAGT TCGATGGAGC CGTTTGTTGC TGAAAGGCAA CGTCACCCTT GGTTCCGAAG GAAATGCCCA TAACATATAT CAGTGGATAT ATATGTGGTT GGTCTACGAC AGACGTCCCG GTAACGTCAA

521 650
MZ054403     TCCCAGGATT GTGGACATAT TTTCTGGGCA ACAGAATAAC CCCGAAACTT GGATGAAGAA TCAGGATAAT GCAGACCGAT TTGTGGTCTG CATGGAGAAA CGGTGTAGTT TGATTGGGCA CGGATATAGC
N1           TCCCAGGATT GTGGACATAT TTTCTGGGCA ACAGAATAAC CCCGAAACTT GGATGAAGAA TCAGGATAAT GCAGACCGAT TTGTGGTCTG CATGGAGAAA CGGTGTAGTT TGATTGGGCA CGGATATAGC
N3           TCCCAGGATT GTGGACATAT TTTCTGGGCA ACAGAATAAC CCCGAAACTT GGATGAAGAA TCAGGATAAT GCAGACCGAT TTGTGGTCTG CATGGAGAAA CGGTGTAGTT TGATTGGGCA CGGATATAGC
N2           TCCCAGGATT GTGGACATAT TTTCTGGGCA ACAGAATAAC CCCGAAACTT GGATGAAGAA TCAGGATAAT GCAGACCGAT TTGTGGTCTG CATGGAGAAA CGGTGTAGTT TGATTGGGCA CGGATATAGC
Consensus    TCCCAGGATT GTGGACATAT TTTCTGGGCA ACAGAATAAC CCCGAAACTT GGATGAAGAA TCAGGATAAT GCAGACCGAT TTGTGGTCTG CATGGAGAAA CGGTGTAGTT TGATTGGGCA CGGATATAGC

651 738
MZ054403     GATACGGCTA TGGTGACCGA TAGATCTCCC AATTTTGCGT ACATGCGATC GCGTCCGATA TCGGTGAACA AGAAATTGAC CGGGACTA
N1           GATACGGCTA TGGTGACCGA TAGATCTCCC AATTTTGCGT ACATGCGATC GCGTCCGATA TCGGTGAACA AGAAATTGAC CGGGACTA
N3           GATACGGCTA TGGTGACCGA TAGATCTCCC AATTTTGCGT ACATGCGATC GCGTCCGATA TCGGTGAACA AGAAATTGAC CGGGACTA
N2           GATACGGCTA TGGTGACCGA TAGATCTCCC AATTTTGCGT -CATGCGATC GCGTCCGATA TCGGTGAACA AGAAATTGAC CGGGACTA
Consensus    GATACGGCTA TGGTGACCGA TAGATCTCCC AATTTTGCGT aCATGCGATC GCGTCCGATA TCGGTGAACA AGAAATTGAC CGGGACTA
```

Supplementary Figure S4

A

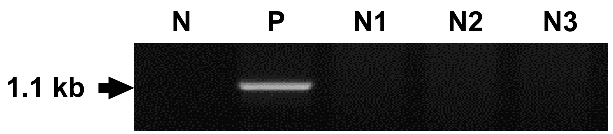

B

Primer	Sequence 5' – 3'	Product Size
pCam-F	GCACTGGAACCCCCAAGC	1.1 kb
pCam-R	TTAGGAACCGGCGGATGCTT	

Supplementary Figure S5

A

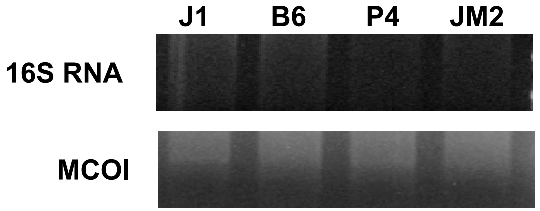

B

Primer	Sequence 5' – 3'	Product Size	Reference
16SRNA-F	CCGGTTTGAACTCAGATCATGT	552 bps	Simon et al. 1994
16SRNA-R	CGCCTGTTTAACAAAAACAT		
MCOI-F	TATATCTTTCCCACGATTAAATAA	1.2 kb	Wang, J. et al. 2011
MCOI-R	GCATATTAATTCTGCCATATTAG		

Supplementary Figure S6A

>MZ054403 Fraxinus symptomless virus

ACTGGCTCGCCCGCGCCGGGGACTTCCGTACGCCCACGTGTCATCATCTTAGGCGTCCACGGAGTGACGCAAATAGT
AGTCGATTTCGATCGACGATCCGTTGTGAAGGACTTTACGTCCTTTAAATACATCTATTTCGTTGTTCATTTCAAAT
GGACTCTTCTAGGAAGAGGAAGTGGTTCGGCGGTCCGGCTAGGACCGCCGGTCGGAAGACTCGAAGAGTGTTGCAGT
TTAACAGCACCCAGAGGAATCAGTTGCCAGCTCAATGGCAACGCGTTCTTCGATATAATCGAAGATCCGGCGGATAC
ACGCCGCCGATGCAGCACATGTCCGATGTGCACGCTATCGAAGACATTGGAGATGGAGGTGTTGTTGTCTGCATCAA
CAACTGTCCCGCCGGCAACAGCGTCGGAACGCGGCATACACAGAAAGTTCGATGGAGCCGTTTGTTGCTGAAAGGCA
ACGTCACCCTTGGTTCCGAAGGAAATGCCCATAACATATATCAGTGGATATATATGTGGTTGGTCTACGACAGACGT
CCCGGTAACGTCAATCCCAGGATTGTGGACATATTTTCTGGGCAACAGAATAACCCCGAAACTTGGATGAAGAATCA
GGATAATGCAGACCGATTTGTGGTCTGCATGGAGAAACGGTGTAGTTTGATTGGGCACGGATATAGCGATACGGCTA
TGGTGACCGATAGATCTCCCAATTTTGCGTACATGCGATCGCGTCCGATATCGGTGAACAAGAAATTGACCGGGACT
ACCTTGTTCAAGGATACCACCGACGGAGGAGTCGGTGATATGGATCGCGGAGCTTATTACTTAGTTTTTAGTACGTT
CCAGTCGTATCATGTAAATGTAACGCTGAATAAGCGAATGTGGTTCAAAAGCATTTAATAAAATGAAATGTATTCAT
TTTCCACTCTCCAGTCTACAATGAGTTTGTTCCATGAACACATTACATCTTGAACACAATACAAGAAGTTGTACAAA
GAAATAACCCCTAAGTTGTCTAATTTTTTATGGATATAAAAATAAAGACGTTGGGATAAATTCCCAGAAATCATCTT
CAACAACGTCTCCTCGTTTATCGTCGTCGACGTCGGTCTTAATGTCGTCCAGATGCGGATAGTCCACCACGCTCTCT
TTATTCCCAATTTCATTCTGAGGTTGTAGTTGAACCTCAGTTCCACGTTGAACATGTGGTGGAACGGTGGATTCGTC
GTCTGTCCAGAGTGTTGGACTCGAATCCAGAACGGGTTGTTCAGTACCCAAGTGTATTCTCCTACTTGTAGTTGCTG
CCATGTGAGTGGCTCCCCTGTGTTGGAATCCATGATTCCGGCACTCTATGGTCTGATGTATTATGCAGCCACACGGC
AGCTCGAGACGTCTAGGCTTGATTACTAGACGCTTCTTCCGTTTGCATCGGTGATGCTTCGCTCCTATAGAAGGGGG
TGTCGGCGATGAAGAATTCTGCATTTGCTAGAGTCCAGAGGTTCAGGCCGTAGTTGCAGTCCAACTGCAGAAAATCT
CTATAAGAAGATTCTCCTGGATTGCCCAGCACGATACCCGGGATACCACCTTTAATTTGAATTGGTTTGGCGTACTT
GGCATTTGATTGCCAATCCCTTTGGGCCCCAATGAGCTCCTTCCAATGTTTCTTTTGGAGATAAGACGGAGTCACGT
CGTCGATGACGTTGTACCAGGCTTTATTGGAGAAAGTTCTAGAATTGAAGTCCATGAAGCCCATTACATAATTATGT
GGGCCTAAAGATCTGGCCCATGCAGTCTTCCCAATCCTGGATTCGCCTTCGAGAATCAGGCTCTTGGGCCTGTGAGG
CCTATCGTTAGTATCTCTGATATTTTCTCTGGCCCAATTTGATAACTGTTCCGGTACCTGGAACGATTCCGGATTGA
ATTCCGGTACATACTCTTTAACTGGTACTGCAAATTCCTTCTCCATGTTAGCTATGATATTATGACGTTGCAGAGCG
TACGTCCTGGGATCGCATCGCTTTATAATTGCGATCGATTCTTCTTTCGTCCTGGCATTAAGAGCTCTTGTGTATTC
CTCGTCGATATCTAGTTTATCGCGACGACGTTTGCGACCGTCCGTCTGGAATTCGCCTTCTTCGACGTAAATTCCTC
CCTTCGCTATGTAGTCGGCGACGTCGTCCGCCGACCTAGCGCTCTGCACGTTCGGATGGTGTACCTCTCCCGTATTT
CGGTCGACGATGTCGAAGAACCTACTGTTCCGACACTTGTACTTCCCTGAGAATTGAATTAGACAGTGGAGGTGCTG
TGCACCGTCGGTGTGCGACTCCTCGCATACCCTTATGAAAACCCAATTGATCGGAAAGCCGAGAGATTTTAACTCGA
TTAAGACGATGTCCTTCGATATCGGACATCGGGCATACGTCAAGAAGTAGTTCTTGGCGTTTATCAAGAATCGCGGT
GAAGATTGGGAAGACATTGGGAATTTGTAAAATTTGGGGATTTTACACAGTGTCGATTGCAAACAGTAACCAGGTCC
TCCTAACTTAAGCCATTTGTATTGGTGACTGGGGACAGAATAATATAGGGTCCTTATTCACTGTTCTCCTACCTTAC
GTCATCATTGACGTCATCAAATTCAAATTCAAATTTCGAATTTTCCCTCCCTACAGTACCCGGGGGTACTATATCTA
TCTTTTCATCAGGCCCCGCAGGGGCTTTATCTCGGGTCCATACCACGGTAGGCCCAATGGGCGAGCCATATAATATT

Supplementary Figure S6B

>MZ054404 Fraxinus symptomless virus

ACTGGCTCGCCCGACCGGGGACTTCCGTACGCCCACGTGTCATCATCTTATCCGTCCACGAAGTGGACGCGAATAGT
AGTCGATTTGGATCGACGATCCGTTTTGGAGAACTTTACGTTCTTTAAATACACCTATTTCGTTGTTCATTTCAAAT
GGACTCTTCTAGGAAGAGGAAGTGGTTCAGCGGTCCGGCTAGGTCCGCCGGTCGGAAGACTCGAAGAGTGTTGCAGT
TTAACAGCACCCAGAGGAATCAGTTGCCAGCTCAATGGCAACGTGTTCTTCGATATAATCGAAGACCCGGCGGATGC
ACGCCGCCGATTCAGCACATGTCCGATGTGCACGCGATCGAAGACATCGGAGATGGCGGTGTTGTTGTCTGCATCAA
CAACTGTCCCGCTGGTAACATCGTCGGAACGCGACATACACAGAAAGTTCGATGGAGCCGTTTGTTGCTGAAAGGCA
ACGTCACCCTCGGTTCCGAAGGAAATGCCCACAATATATATCAGTGGATATATATGTGGTTAGTCTACGATAGACGT
CCTGGTAACGTCAATCCCAGGATTATTCGATTATTTTCTGGGCAACAGAATAACCCCGAAACTTGGATGAAGAATCA
GGATAATGCAGACCGATTTGTGGTCTGCATGGAGAAACGGTATAGTTTGATTGGGCACGGATATAGCGATACGGCTA
TGGTGACCGATAGATCTCCCAATTTTGCTTACATGCGATCGCGTCCGATATCGGTGAACAAGAAACTGACTGGGACT
ACCTTGTTCAAGGATACCACCGACGGAGGAGTCGGTGATATGGATCGCGGAGCTTATTACTTAGTTTTTAGTACGTT
CCAGTCGTATCATGTAAATGTAACGCTGAATAAGCGAATGTGGTTCAAAAGCGTTTAATAAAATGAAATGTATTCAT
TTTCCACTCTCCAGTCTACAATGAGTTTGTTCCATGAACACATTACATCGTGAACACAATACAAGGAGTTGTACAAA
GAAATAACCCCTAAGTTGTCTAATTTTTTATGGATATAAAAATAAAGACGTTGGGATAAATTCCCAGAAATCATCTT
CAACAACGTCTCCTCGTTTATCGTCATCGACGTCGGTCTTAATGTCGTCCAGATGCGGATAGTCCACCACGCTCTCT
TTATTCCCAATTTCATTCTGAGGTTGTAGTTGAACCTCAGTTCCACGTTGAACATTTGGTGGAACGGTGGATTCGTC
GTCTGTCCAGAGTGTTGGACTCGAATCCAGAACGGGTTGTTCAATACCCAAGTGTATTCTCCGACTTGTAGTTGCTG
CCATGTGAGTGGCTCCCCTGTGTTGGAATCCATGATTCCGGCACTCTATTGTCTGATGTATTATGCAGCCACACGGC
AGTTCGAGACGTCTAGGCTTGATGACTAGACGCTTCTTCCGTTTGCATCGGTGATGCTTCGCTCCTATAGAAGGGGG
TGTCGGCGATGAAGAATTCTGCATTTGCTATAGTCCAGAGATTCAAGCCGTAGTTGCAGTCTAACTGCAAAAAATCT
CTATAAGAAGATTCTCCTGGATTGCACAGCACGATACACGGGATACCACCTTTAATTTGAATTGGTTTGCCGTACTT
GGCATTTGATTGCCAATCCCTTTGGGCCCCAATGAGCTCCTTCCAATGTTTCTTTTGGAGATAAGACGGAGTGACGT
CGTCGATGACGTTGTACCAGGTTTTATTGGAGAACGTTTTACAATTGAAGTCCATGAAGCCCATTACATAATTATGT
GGGCCTAAAGATCTGGCCCAAGCAGTTTTCCCAAGCCTGGATTCGCCTTCGAGGATCAGACTTTTGGGCCTGTGAGG
CCTATCGTTAATATCTCTGATATTTTCTCTGACCCATATTGATAACTGTTCGGGTACCTGGAATGACTCCGGATTGA
ACTCCGGTGCATATTCTTGAACAGGTGGTGCAAATTCCTTCTCCATATTAGCAATGATATTGTGACGTTGCAGAGCG
TACGTCCTGGGATCGCATCGCTTTATAATTGCGATCGATTCTTCTTTCGTTCTGGCATTTAGAGCTCTTGTGTATTC
CTCGTCGAGATCTGTTTTATCGCGACGACGTTTGCGACCGTCCGCCCGAAACTCGCCTTCTTCGACGTAAATTCCTC
CCTTCGCTATGTAGTCGGCGACGTCGTCCGCCGACCCCGCGCTCTGCACGTTCGGATGGTACACCTCTCCTGTATTT
CGGTCGACTATGTCGAAGAACCTACTGTTCCGACACTTGTACTTCCCTGAGAATTGAATTAGACAGTGGAGGTGCTG
TGCACCGTCGGTGTGCGACTCCTCGCATACCCTTATGAAAACCCAATTGATCGGAAAGCCGAGCGATTTTAACTCGA
TTAAGACGATGTCCTTCGATATGGGACATCGGGCATACGTCAAGAAGTAGTTCTTGGCGTTAATTAAGAAACGCGGT
GACGATTGGGAAGACATTGGGGATTTGTAAAATTTGGGGATTTTACACAGTGCCAATTGAAAACAGTAACAGGGGCC
TTCAATATAACCTATTTGTATTGGTGACTGGGGACAGTGTTATATAGGGTCCTTATTCACTGTTCTCTTACCTTACG
TCATCATTGACGTCATCACATTCAAATTTGAATTTTCCCTCCCTACAGTACCGGGGGTACTATATCTATCATTTCAT
CAGGCCCCGCAGGGGCTTTATCTCGGGTCCATACCACGGTAGACCCAATAGGCGAGCCATATAATATT

Supplementary TableS1. Primers used to detect FSMV DNA-A. DNA-B and to confirm the existence of virion-sense (VS) and complementary-sense (CS) DNA molecules in strand-specific amplification PCR procedure.

Primer	Sequence (5'-3')	Loci	Size
Ash gemini 2F	CCACGTGTCATCATCTTAGG	27 - 46	737bp
Ash gemini 2R	TAGTCCCGGTCAATTTCTTG	745 - 764	

Primer	Sequences 5'-3'	Product
**Tree-F2**	AGT GTT GGA CTC GAA TCC AGA A	2.7 kb
**Tree-R2**	CTG GAC AGA CGA CGA ATC CA	

Primer	Sequences 5'-3'	Product
**PBL1v20140**	GCCTCTGCAGCARTGRTCKATCTTCATACA	600 bps
**PCRc1**	CTAGCTGCAGCATATTTACRWATGCCA	

Primer	Sequences 5'-3'
**Taq**	AGTTTAAGAACCCTTCCCGC
**OVS**	GGAGGTGTTGTTGTCTGCATC
**OCS**	GTCTGTCGTAGACCAACCACA
**Taq-OVS**	AGTTTAAGAACCCTTCCCGCG<br>GAGGTGTTGTTGTCTGCATC
**Taq-OCS**	AGTTTAAGAACCCTTCCCGCGT<br>CTGTCGTAGACCAACCACA
